# Supplementary material for: Content-rich biological network constructed by mining PubMed abstracts
Source: BMC Bioinformatics. 2004 Oct 8;5:147. doi: 10.1186/1471-2105-5-147 (PMC528731; doi:10.1186/1471-2105-5-147)
Supplement: Additional File 2 — The original results of the above study (non-essential files are deleted to keep the file size under the limit set by BMC bioinformatics). [file 1471-2105-5-147-S2.bz2 › chilibotAdditionalFile2/dip40/574ID7703225E10941/html/NTF3_BDNF.html]

 


 **NTF3** and **BDNF** 
  
Found 1139 abstracts in PubMed, retrieved 40.  
 

 What does Google say? 
 PDF only 
| .edu only 

---

**Interactive relationship** (e.g. stimulation, inhibition, etc)

**Neutral relationship**- In the gustatory system, NT3  [ **NTF3** ]  is unable to replace the actions of  **BDNF**  possibly because of a temporally selective expression of TrkB in taste neurons.  Ref: 12620975 Development< MedlineTA>Development,
- AAV  **BDNF**  and AAV NT 3  [ **NTF3** ]  transduced 293 human kidney cells produced and secreted  **BDNF**  or NT 3  [ **NTF3** ] , respectively, in vitro.  Ref: 12676157 Neuroscience, 2003
- Analysis of  **BDNF**  mice showed striking differences in the ability of NT3  [ **NTF3** ]  to promote survival, short range innervation and synaptogenesis in different sensory systems.  Ref: 12620975 Development< MedlineTA>Development, 2003

**Non-interactive relationship** (e.g. studied together, co-existance, homology, etc.)

- In the present study we have replaced the coding part of the  **BDNF**  gene in mice with that of NT3  [ **NTF3** ]  to analyse the specificity and selective roles of  **BDNF**  and NT3  [ **NTF3** ]  during development.  Ref: 12620975 Development< MedlineTA>Development, 2003
- The effect of HAEC CM was significantly higher than that of  **BDNF** , neurotrophin 3  [ **NTF3** ]  and CNTF.  Ref: 12676329 Neurosci Lett, 2003
- Continuous low dose treatment with brain derived neurotrophic factor  [ **BDNF** ]  or neurotrophin 3  [ **NTF3** ]  protects striatal medium spiny neurons from mild neonatal hypoxia ischemia a stereological study.  Ref: 12732247 Neuroscience< MedlineTA>Neuroscience, 2003
- We found that 1 trk C, a receptor for neurotrophin 3  [ **NTF3** ]  NT 3  [ **NTF3** ] , is dominantly expressed in RCEC, but trk B, a receptor for brain derived neurotrophic factor  [ **BDNF** ] , is dominantly expressed in RAEC.  Ref: 12745089 Biochem Biophys Res Commun, 2003
- Neurotrophin 3  [ **NTF3** ]  and brain derived neurotrophic factor  [ **BDNF** ]  in the gastrocnemius muscle kept constant expression in mRNA and protein during the postnatal period.  Ref: 12657452 Neurosci Res, 2003
- Cultured Xenopus spinal cord neurons were treated with a mixture of  **BDNF** , glia derived neurotrophic factor, NT 3  [ **NTF3** ] , and NT 4, in addition to forskolin and IBMX or the cell permeant form of cAMP, to elevate the cAMP level.  Ref: 12832528 J Neurosci, 2003
- LR or DR alter the expression of the mRNAs for NGF,  **BDNF**  and NT3  [ **NTF3** ]  and CNTF within the visual system.  Ref: 12855194 Brain Res Dev Brain Res, 2003
- NGF,  **BDNF** , NT3  [ **NTF3** ] , and NT4 were localized in the lacrimal gland acini with differing cellular distributions.  Ref: 12882781 Invest Ophthalmol Vis Sci, 2003
- Our results suggest that overexpression of  **BDNF** , NT 3  [ **NTF3** ] , trkB and trkC in motoneuron areas neighboring the site of mechanical compression may represent compensatory changes in response to the compromised neuronal function at the level of compression,.  Ref: 12774239 Acta Neuropathol Berl , 2003
- Other measures of neuroprotection such as brain weight and striatal infarct volume were also undertaken. **BDNF**  or NT 3  [ **NTF3** ]  significantly increased the total number of surviving medium spiny neurons by 43% and 33% respectively.  Ref: 12732247 Neuroscience< MedlineTA>Neuroscience, 2003
- In all studied species both NGF and NT 3  [ **NTF3** ]  like immunoreactivity IR were present in the enteric nervous system, while  **BDNF**  like IR was never detected.  Ref: 12809982 Neurosci Lett, 2003
- Compared with controls, a modest though significant improvement in hind limb function was found in rats that received AAV  **BDNF**  and AAV NT 3  [ **NTF3** ] .  Ref: 12676157 Neuroscience, 2003
- To foster axonal growth from a Schwann cell bridge into the caudal spinal cord, spinal cells caudal to the implant were transduced with adeno associated viral AAV vectors encoding for brain derived neurotrophic factor  [ **BDNF** ]   **BDNF**  and neurotrophin 3  [ **NTF3** ]  AAV NT 3  [ **NTF3** ] .  Ref: 12676157 Neuroscience, 2003
- We first examined the relative potency of the neurotrophins, brain derived neurotrophic factor  [ **BDNF** ]   **BDNF** , neurotrophin 3  [ **NTF3** ]  and nerve growth factor to prevent glutamate mediated apoptosis.  Ref: 12829403 Neurotox Res, 2002
- We found no evidence for growth of regenerated axons from the Schwann cell implant into the caudal cord.Our results suggest that AAV vector mediated overexpression of  **BDNF**  and NT 3  [ **NTF3** ]  in the cord caudal to a Schwann cell bridge modified the local lumbar axonal circuitry, which was beneficial for locomotor function.  Ref: 12676157 Neuroscience, 2003
- We demonstrate that mRNA for brain derived neurotrophic factor  [ **BDNF** ]   **BDNF** , but not neurotrophin 3  [ **NTF3** ]  or nerve growth factor NGF, is readily detectable in PBMC.  Ref: 12753507 Scand J Immunol, 2003
- Here, we examine how pulpal cells and 3T3 fibroblasts overexpressing certain neurotrophic factors nerve growth factor, brain derived neurotrophic factor  [ **BDNF** ] , neurotrophin 3  [ **NTF3** ] , neurotrophin 4, glial cell line derived neurotrophic factor or neurturin influence survival and growth of single trigeminal ganglion neurones in vitro in quantitative terms.  Ref: 12770558 Neuroscience, 2003
- As the VMN undergoes sexual differentiation, we compared  **BDNF** , NGF, NT 3  [ **NTF3** ] , and trkB mRNA expression in the VMN in males and females at embryonic day 20 and PND 4, but found no differences between them.  Ref: 12824057 Brain Res Mol Brain Res, 2003
- RESULTS Expression of NT4 was detected in the inner and outer nuclear layers of all the retinal sections examined but no NT3  [ **NTF3** ]  and  **BDNF**  staining was seen.  Ref: 12642316 Br J Ophthalmol, 2003
- Immunohistochemistry was performed to investigate the pattern of staining of NT3  [ **NTF3** ] , NT4,  **BDNF** , GFAP, and CD68 in 7 mm sections of fixed specimens.  Ref: 12642316 Br J Ophthalmol, 2003
- Ribonuclease protection assay of cultured OEC revealed expression of NGF,  **BDNF** , GDNF, and CNTF mRNA, while NT3  [ **NTF3** ]  and NT4 mRNA were not detectable.  Ref: 12684030 Exp Neurol, 2003
- To determine which of the neurotrophins NTs nerve growth factor NGF, brain derived neurotrophic factor  [ **BDNF** ]   **BDNF** , neurotrophin 3  [ **NTF3** ]  NT3  [ **NTF3** ] , and neurotrophin 4 5 NT4 and their receptors NTrs, TrkA, TrkB, TrkC, and p75, are present in the adult rat lacrimal gland.  Ref: 12882781 Invest Ophthalmol Vis Sci, 2003
- However, despite overlapping expression of TrkB and TrkC in many sensory ganglia, brain derived neurotrophic factor  [ **BDNF** ]  and neurotrophin 3  [ **NTF3** ]  null mutant mice display selective losses in neuronal subpopulations.  Ref: 12620975 Development< MedlineTA>Development, 2003
- The expression pattern of neurotrophins changes with age, with falling NGF, NT3  [ **NTF3** ]  and NT4 but increasing granule cell  **BDNF** .  Ref: 12879965 Cerebellum, 2002
- We recently found that human amniotic epithelial HAE cells secrete biologically active neurotrophins such as brain derived neurotrophic factor  [ **BDNF** ]  and neurotrophin 3  [ **NTF3** ] , both of which exhibit trophic activities on dopamine DA neurons.  Ref: 12865158 Brain Res, 2003
- Stored NGF,  **BDNF**  and NT 3  [ **NTF3** ]  was demonstrated by Western blotting and flow cytometry.  Ref: 12752594 Clin Exp Allergy, 2003
- For this purpose, serial immunohistochemical and immunoblot analyses were performed for the expression levels of endogenous brain derived neurotrophic factor  [ **BDNF** ]   **BDNF** , neurotrophin NT 3  [ **NTF3** ] , and their receptors, trkB and trkC in 24 tip toe walking Yoshimura mice twy twy aged 12 24 weeks.  Ref: 12774239 Acta Neuropathol Berl , 2003
- Neurotrophin levels were determined by using brain derived neurotrophic factor  [ **BDNF** ]   **BDNF**  and neurotrophin 3  [ **NTF3** ]  NT3  [ **NTF3** ]  ELISA.  Ref: 12621105 Pediatr Res< MedlineTA>Pediatr Res, 2003
- The proteins of the mammalian neurotrophin family nerve growth factor NGF, brain derived neurotrophic factor  [ **BDNF** ]   **BDNF** , neurotrophin 3  [ **NTF3** ]  NT 3  [ **NTF3** ]  and neurotrophin 4 5 NT 4 5 were originally identified as neuronal survival factors.  Ref: 12787574 Prog Neurobiol, 2003
- Nerve growth factor, brain derived neurotrophic factor  [ **BDNF** ]   **BDNF**  and neurotrophin 3  [ **NTF3** ]  NT 3  [ **NTF3** ]  mRNAs were revealed in OLGs in vivo and in culture.  Ref: 12843289 J Neurosci, 2003
- AIMS To investigate the staining pattern of neurotrophin 3  [ **NTF3** ]  NT3  [ **NTF3** ] , neurotrophin 4 NT4, and brain derived neurotrophic factor  [ **BDNF** ]   **BDNF**  as well as glial fibrillary acid protein GFAP and CD68 in lasered human retina.  Ref: 12642316 Br J Ophthalmol, 2003
- BY MEANS OF IMMUNOCHEMISTRY AND IMMUNOHISTOCHEMISTRY, WE INVESTIGATED IN THE GUT OF TELEOSTEAN SPECIES THE PRESENCE AND LOCALIZATION OF THREE NEUROTROPHINS nerve growth factor NGF, brain derived neurotrophic factor  [ **BDNF** ]   **BDNF**  and neurotrophin NT 3  [ **NTF3** ] .  Ref: 12809982 Neurosci Lett, 2003
- NGF,  **BDNF** , NT 3  [ **NTF3** ] , NT 4 5 and NT 6 constitute NGF family the neurotrophins, which may enhance both survival and differentiation of neurons from the EGF responsive hippocampal and the subventricular zone neural stem cells.  Ref: 12650066 Sheng Li Ke Xue Jin Zhan, 2002
- In contrast, the expression levels of  **BDNF** , NT 3  [ **NTF3** ] , trkB and trkC were significantly higher at the rostral and caudal sites immediately adjacent to the maximal compression site.  Ref: 12774239 Acta Neuropathol Berl , 2003
- The pump continuously infused either  **BDNF**  4.5 & ; mgr ; g day, NT 3  [ **NTF3** ]  12 & ; mgr ; g day, or vehicle solution into the right striatum for 3 days from postnatal day 7.  Ref: 12732247 Neuroscience< MedlineTA>Neuroscience, 2003
- This study aimed to investigate whether continuous, low dose, intracerebral infusion of either brain derived neurotrophic factor  [ **BDNF** ]   **BDNF**  or neurotrophin 3  [ **NTF3** ]  NT 3  [ **NTF3** ]  could protect against striatal neuronal loss in mild neonatal hypoxic ischaemic brain injury.  Ref: 12732247 Neuroscience< MedlineTA>Neuroscience, 2003
- Prior exposure to MeHg had no effect on NT 3  [ **NTF3** ]  or  **BDNF**  stimulated PI hydrolysis in the cerebellum.  Ref: 12835095 Neurotox Res, 2000
- Cultured cortical progenitors express  **BDNF**  and NT 3  [ **NTF3** ] , as well as their receptors TrkB tyrosine kinase receptor B and TrkC.  Ref: 12832539 J Neurosci, 2003
- Here, we investigated the role of two endogenously produced growth factors, the neurotrophins brain derived neurotrophic factor  [ **BDNF** ]  and neurotrophin 3  [ **NTF3** ]  NT 3  [ **NTF3** ] , in the early progenitor to neuron transition.  Ref: 12832539 J Neurosci, 2003
- Geniculate neurons cultured with either  **BDNF**  or NT4 had similar passive membrane and action potential properties, but these characteristics were significantly different from those of neurons cultured with NGF or NT3  [ **NTF3** ] .  Ref: 12676146 Neuroscience, 2003
- In the following study, we determined the effects of glutamate on early passaged rat Müller cells, specifically their expression of neurotrophic factors including  **BDNF** , nerve growth factor, neurotrophin 3  [ **NTF3** ] , neurotrophin 4, and glial cell line derived neurotrophic factor.  Ref: 12654519 Brain Res Mol Brain Res, 2003
- We assessed basal levels of several substances which may be involved in differential temporal ethanol vulnerability in neonatal cerebellum, and analyzed alterations in these substances after early ethanol exposure.METHODSAssessments were made of neurotrophic factors nerve growth factor, brain derived neurotrophic factor  [ **BDNF** ] , neurotrophin 3  [ **NTF3** ] , and neurotrophin 4.  Ref: 12711928 Alcohol Clin Exp Res, 2003
- Moreover, trophic effects of OLG conditioned medium were partially blocked by K252a, a trk tyrosine kinase inhibitor, and by neutralizing anti  **BDNF**  or anti NT 3  [ **NTF3** ]  antisera, indicating that neurotrophins may mediate these effects, perhaps in concert with other signals.  Ref: 12843289 J Neurosci, 2003
- OBJECTIVES The aim of this study was to assess whether eosinophils of allergic and non allergic donors produce, store and release the neurotrophic factors NGF,  **BDNF**  and NT 3  [ **NTF3** ] .  Ref: 12752594 Clin Exp Allergy, 2003
- Retrograde tracing demonstrated that twice as many neurons with processes extending toward the Schwann cell graft were present in the second lumbar cord segment of AAV  **BDNF**  and AAV NT 3  [ **NTF3** ]  injected animals compared with controls.  Ref: 12676157 Neuroscience, 2003
- In this work the expression and anatomical localization of NGF,  **BDNF**  and NT 3  [ **NTF3** ]  and of corresponding high affinity receptors TrkA, TrkB full length and truncated TR isoforms, TrkC and of the low affinity p75 receptor were assessed in surgical samples from adult human lung by reverse transcriptase polymerase chain reaction RT PCR, Western blot and immunohistochemistry.  Ref: 12791675 Am J Respir Cell Mol Biol< MedlineTA>Am J Respir Cell Mol Biol, 2003
- In the retina, superior colliculus SC, primary visual cortex V1, hippocampus HIPP and cerebellum CBL, using a ribonuclease protection assay RPA, we examined expression of the mRNAs for nerve growth factor NGF,  **BDNF** , NT3  [ **NTF3** ] , NT4, ciliary neurotrophic factor CNTF and glial cell line derived neurotrophic factor GDNF.  Ref: 12855194 Brain Res Dev Brain Res, 2003
- Neurons secreted the brain derived neurotrophic factor  [ **BDNF** ]  and neurotrophin 3  [ **NTF3** ]  to induce neuron differentiation, while astrocytes secreted ciliary neurotrophic factor for astrocyte differentiation.  Ref: 12837625 Mol Cell Neurosci, 2003
- Increased neuron losses associated with the presence of truncated TrkB were most severe in regions where neuron survival is most dependent on brain derived neurotrophic factor  [ **BDNF** ]  and neurotrophin 3  [ **NTF3** ] .  Ref: 12654337 Neuroscience, 2003
- The intrastriatal presence of  **BDNF**  or NT 3  [ **NTF3** ]  was verified immunohistochemically.  Ref: 12732247 Neuroscience< MedlineTA>Neuroscience, 2003
- Immunoreactivities for  **BDNF** , NT 3  [ **NTF3** ] , trkB and trkC were preferentially localized in the gray matter, particularly in the anterior horn cells.  Ref: 12774239 Acta Neuropathol Berl , 2003
- Our study showed that while glutamate treatment did not promote cell death, it upregulated secretion of  **BDNF** , NGF, NT 3  [ **NTF3** ] , NT 4, and GDNF by Müller cells.  Ref: 12654519 Brain Res Mol Brain Res, 2003
- Western blot analysis confirmed these results, showing that the lacrimal gland expresses NGF,  **BDNF** , NT3  [ **NTF3** ] , and NT4 as well as the NTrs TrkA, TrkB, and TrkC and the p75 protein.  Ref: 12882781 Invest Ophthalmol Vis Sci, 2003
- Acute exposure to 10 micro M MeHg increased basal PI hydrolysis in cortical slices and increased NT 3  [ **NTF3** ]  and  **BDNF**  stimulated PI hydrolysis in slices from the cerebellum.  Ref: 12835095 Neurotox Res, 2000
- RT PCR showed positive bands at the appropriate sizes for NGF,  **BDNF** , NT3  [ **NTF3** ] , and NT4, and for the receptors TrkA, TrkB, TrkC, and p75.  Ref: 12882781 Invest Ophthalmol Vis Sci, 2003
- The production, storage and release of the neurotrophins nerve growth factor, brain derived neurotrophic factor  [ **BDNF** ]  and neurotrophin 3  [ **NTF3** ]  by human peripheral eosinophils in allergics and non allergics.  Ref: 12752594 Clin Exp Allergy, 2003
- In addition, in order to clarify the differerences in the results obtained with the various neurotrophins in different animal preparations we also report new data on the action of NGF,  **BDNF** , NT3  [ **NTF3** ]  and NT4 in the same preparation, namely the visual cortex of the rat.  Ref: 12671228 Restor Neurol Neurosci< MedlineTA>Restor Neurol Neurosci, 2003
- CONCLUSIONS Eosinophils store, produce and release NGF,  **BDNF**  and NT 3  [ **NTF3** ] .  Ref: 12752594 Clin Exp Allergy, 2003
- We have examined the chemokinetic effects of nerve growth factor NGF, brain derived growth factor  **BDNF** , and neurotrophin 3  [ **NTF3** ]  NT 3  [ **NTF3** ]  on Schwann cells in vitro using a chemotaxis chamber.  Ref: 12625389 Scand J Plast Reconstr Surg Hand Surg, 2003
- Interestingly, when a complete course of antenatal steroids was given,  **BDNF**  and NT3  [ **NTF3** ]  cord blood levels were higher than when no steroid was given.  Ref: 12621105 Pediatr Res< MedlineTA>Pediatr Res, 2003
- Neurotrophin 3  [ **NTF3** ]  NT 3  [ **NTF3** ]  and brain derived neurotrophic factor  [ **BDNF** ]   **BDNF**  stimulated PI hydrolysis in neocortical and cerebellar slices from neonatal rats in a concentration dependent manner 30 1000 ng ml.  Ref: 12835095 Neurotox Res, 2000
- Effects of three neurotrophins, i.e., nerve growth factor, brain derived neurotrophic factor  [ **BDNF** ] , and neurotrophin 3  [ **NTF3** ] , on the expression of four neurotransmitter synthesizing enzymes, i.e. choline acetyltransferase ChAT, tyrosine hydroxylase TH, dopamine beta hydroxylase DBH, and glutamate decarboxylase 65 were investigated in cultured mouse neural stem cells.  Ref: 12633895 Neurosci Lett< MedlineTA>Neurosci Lett, 2000
- Ganglia were explanted from rats at gestational day 16, when growing neurites have entered the papilla core, and maintained in culture with added brain derived neurotrophic factor  [ **BDNF** ]   **BDNF** , neurotrophin 4 NT4, nerve growth factor NGF or neurotrophin 3  [ **NTF3** ]  NT3  [ **NTF3** ] .  Ref: 12676146 Neuroscience, 2003
- Messenger RNAs encoding other neurotrophins, including nerve growth factor NGF and neurotrophin 3  [ **NTF3** ]  NT 3  [ **NTF3** ]  and the  **BDNF**  receptor trkB, were not selectively localized in neonatal VMN.  Ref: 12824057 Brain Res Mol Brain Res, 2003
